# Vorinostat (SAHA) and Breast Cancer: An Overview

**DOI:** 10.3390/cancers13184700

**Published:** 2021-09-19

**Authors:** Anna Wawruszak, Lidia Borkiewicz, Estera Okon, Wirginia Kukula-Koch, Syeda Afshan, Marta Halasa

**Affiliations:** 1 Department of Biochemistry and Molecular Biology, Medical University of Lublin, 20-093 Lublin, Poland; lidia.borkiewicz@umlub.pl (L.B.); estera.okon@umlub.pl (E.O.); marta.halasa@umlub.pl (M.H.); 2 Department of Pharmacognosy, Medical University of Lublin, 20-093 Lublin, Poland; wirginiakukulakoch@umlub.pl; 3 Institute of Biomedicine and FICAN West Cancer Centre, University of Turku, 20521 Turku, Finland; syeda.afshan@utu.fi

**Keywords:** breast cancer, vorinostat (SAHA), suberoylanilide hydroxamic acid, histone deacetylase inhibitor (HDI), histone acetylation, histone deacetylases (HDACs), epigenetics, targeted therapy

## Abstract

**Simple Summary:**

Breast cancer (BC) is the most frequent malignancy diagnosed in 2020 worldwide. Despite significant advances in BC therapy, its pathogenesis is still not fully understood, and effective therapy is one of the most important challenges in current oncology. The article presents the state of the knowledge on vorinostat (SAHA) in the therapy of various histological subtypes of BC, individually or in polytherapy with other active compounds, in in vitro, in vivo and clinical trials settings.

**Abstract:**

Vorinostat (SAHA), an inhibitor of class I and II of histone deacetylases, is the first histone deacetylase inhibitor (HDI) approved for the treatment of cutaneous T-cell lymphoma in 2006. HDIs are promising anticancer agents that inhibit the proliferation of many types of cancer cells including breast carcinoma (BC). BC is a heterogeneous disease with variable biological behavior, morphological features, and response to therapy. Although significant progress in the treatment of BC has been made, high toxicity to normal cells, serious side effects, and the occurrence of multi-drug resistance limit the effective therapy of BC patients. Therefore, new active agents which improve the effectiveness of currently used regimens are highly needed. This manuscript analyzes preclinical and clinical trials data of SAHA, applied individually or in combination with other anticancer agents, considering different histological subtypes of BC.

## 1. Introduction

Recent studies indicate that non-communicable diseases (NCDs) are responsible for more than 75% of premature deaths. Statistics show that out of every 10 NCD deaths, four are due to cardiovascular disease, and three are due to cancer [1]. In 2020, 19.3 million new cancer cases were diagnosed, and 10 million deaths were noted worldwide. Cancer incidence and, ultimately, mortality is expeditiously increasing. It may be partly explained by the aging and increase in the number of a population as well as socio-economic development. According to the World Health Organization (WHO), the number of female breast cancer (BC) cases has overtaken lung cancer. BC is the most commonly diagnosed among all cancer types (11.7% of all cancer cases) now, with a mortality rate of 6%. BC accounts for 1 in 4 cases of cancer and 1 in 6 cases is fatal, which exhibits a great need for an effective diagnosis system and anticancer therapy [2].

About 20% of all BC cases have family origins, presenting etiological independence to the specific set of genes, which predisposes BC [3]. The most common non-genetic risk factors have a hormonal background, including gender, reproduction status, breastfeeding, and menopause. Predominantly, non-genetic BC is diagnosed among menopausal women with high expression of estrogen receptor (ER) [4]. BC subtypes differ due to histological and molecular profiles, behaviors, and response to treatment. The direction of therapy is chosen based on tumor morphology, its grade and size, the presence of metastases to the lymph nodes, as well as gene expression of BC critical markers: ER, progesterone receptor (PR), and human epidermal growth factor receptor 2 (HER2) [5].

The BC is a heterogeneous disease at the molecular and clinical levels. The main classification of BC includes two groups based on the hormone receptors and other proteins involved in cancer development and progression (Table 1).

BC comprises five molecular-based intrinsic subtypes: luminal A, luminal B, HER2-enriched, triple-negative breast cancer (TNBC), and normal-like subtype. ER-positive (ER+) BC subtypes include a set of genes correlated with luminal cells, including luminal cytokeratins (e.g., cytokeratin 8 and 18) [10]. ER+ cancers are divided into two main subclasses: luminal A and luminal B. These subtypes can differ due to HER2 expression and cell proliferation index. Luminal A cancers express lower histological grade and slowly grow, indicating the best prognosis for patients, with a meaningfully lower relapse rate than other subtypes [11]. Normal-like BC is similar to luminal A, expressing the same set of clinical markers; however, the prognosis is slightly worse than the luminal A subtype. Luminal B cancers express a lower level of ER-related genes and a higher level of proliferation-related and growth factor receptor signaling genes. Additionally, luminal B tumors may be accompanied by HER2 gene amplification and/or its overexpression. They tend to grow faster and are more aggressive than luminal A cancers [12]. The next subtype of BC is HER2-enriched/overexpressed BC, which expresses neither ER nor PR, but is HER2-positive. HER2-enriched BC is defined by high expression of growth factor receptor signaling genes as well as cell cycle-related genes, and low expression of estrogen-related and basal-related genes [13]. This BC subtype grows faster and indicates worse patients’ prognosis compared to both luminal subtypes [5]. Unlike luminal and HER2-enriched tumors, TNBC subtype accounts for 12.5% of all BC cases, does not express any molecular receptors (ER, PR, HER2). This subtype is highly aggressive and indicates poor patients’ prognosis. The relapse rate in TNBC patients is higher compared to other subtypes. Due to the lack of critical molecular targets, the treatment of TNBC is very limited and chemotherapy remains the first choice of therapy [14,15,16].

Currently, in patients with diagnosed BC different treatment strategies are implemented, including surgery, chemotherapy, radiotherapy as well as targeted, combined, and hormonal therapy [17,18]. For women with early-stage of BC, the standard treatment is breast-conserving surgery combined with radiotherapy or mastectomy. Additionally, adjuvant systemic therapy is applied for nonmetastatic BC patients [19]. The type of systemic therapy is associated with the BC subtype. The endocrine treatment is dedicated to patients with hormone receptor-positive (HR+) tumors. In some cases, endocrine therapy is supported by chemotherapy, whereas patients with triple-negative tumors receive chemotherapy alone [20]. Although the BC therapies are constantly developed and new personalized therapies are being proposed continuously [21,22], there is still a need to find out better optimizations for the currently implemented treatment regimens to overcome serious side effects, and ultimately improve patients’ outcomes. A better understanding of the molecular landscape of BC allows for the better possibility of implementation of sufficient treatment, including targeted or immunotherapy as well as a combined therapy.

## 2. Epigenetic Basis of Cancer

**Figure 1:**
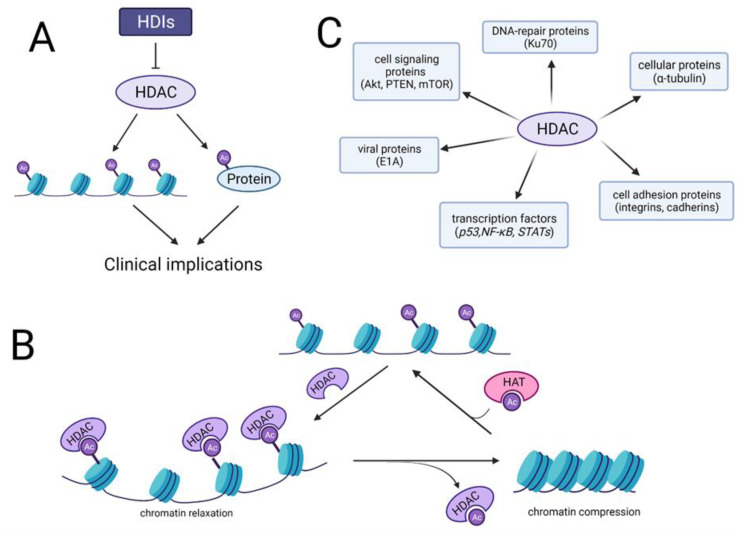
(**A**) Histone deacetylase inhibitors (HDIs) block HDACs expression leading to upregulation of acetylation histone and non-histone protein what resulted in clinical implications. (**B**) Reversible posttranslational lysine residues acetylation of histones is maintained by HATs and HDACs. Removal of acetyl group by HDACs leads to condensation of chromatin and its inactivation. (**C**) The multiple roles of HDAC enzymes in cells. Regulation of non-histone proteins (transcription factors, adhesion proteins, cellular proteins, DNA-repair proteins, cell signaling and viral proteins) according to their acetylation state, which indicates that HDACs can influence a multitude of physiological pathways in different cells.

Molecularly, cancer is defined by uncontrolled cells division, which is following by genetic alterations [23] and epigenetic modifications [24]. The activation of oncogenes and deactivation of tumor suppressor genes results in inhibition or inactivation of apoptotic signaling pathways, thus increasing cell proliferation [23,25]. Epigenetic modifications, including histone and non-histone modifications, DNA methylation and microRNAs regulation, significantly affect cell cycle progression and apoptosis without modifying the gene sequence. However, the silencing of cancer-related genes by epigenetic modifications is believed to be a critical mechanism in tumor formation. Unlike genetic modifications, epigenetic changes are reversible. Epigenetic maintenance provides eukaryotic genome stability through affecting key biological signaling pathways [26]. Many studies currently focus on signaling pathways associated with reversible acetylation of histone and non-histone proteins, as it could be considered a critical tumor marker in many cancer types [27]. Abnormal reversible histone acetylation is also closely related to BC, initiating molecular signaling pathways involved in tumor progression [28,29]. Reversible acetylation is maintained by two groups of enzymes: histone acetyltransferases (HATs) and histone deacetylases (HDACs) (Figure 1A,B) [30]. In addition to chromatin remodeling, HDACs affect non-histone proteins at specific sites, including tumor suppressors [31,32,33] and oncoproteins [34,35] involved in tumor signaling pathways, ultimately affecting cell fate [36]. HDACs deacetylate many groups of non-histone proteins including: transcription factors [37,38,39], cell adhesion proteins [40,41], cellular proteins [42], DNA repair proteins [43], cell signaling proteins [44,45], and viral proteins [46] (Figure 1C). However, HDACs role in tumorigenesis is controversial: either promoting cancer cell survival or causing cell death among different types of cancers [47,48,49,50].

The 18 members of the HDAC family are divided into four groups due to their sequence similarity to yeast counterparts as well as catalytic dependence. The first group consists of four members, including HDAC1/2/3 and 8. The second group is divided into two subgroups: IIa, including HDAC4/5/7/9; and IIb, including HDAC6/10, while the fourth group consists of only one member: HDAC11 [51]. All HDACs mentioned above require a zinc ion to their catalytic activity. The third group of HDACs called sirtuins consists of 7 members: SIRT1-SIRT7, and unlikely to rest of HDACs members they require nicotinamide adenine dinucleotide (NAD) to their catalytic activity [52]. HDACs family members are highly expressed in BC [53,54,55]; therefore, their modulators are in demand.

## 3. Histone Deacetylase Inhibitors (HDIs)

HDACs play as transcription repressors by promoting chromatin condensation through histone deacetylation. Histone deacetylase inhibitors (HDIs) selectively modulate gene transcription through the changes in the structure of proteins involved in transcriptional machinery [56,57,58]. Besides altering gene transcription, HDACs affect non-histone targets, including transcriptional factors [31,32,59,60,61], hormonal receptors [62,63,64] as well as signaling mediators [65,66]. These targets could also be modulated by HDIs. Although the modulation of HDACs expression is a promising tool in anticancer treatment, only a few HDIs have already been approved by the United States Food and Drug Administration (FDA) for cancer treatment [67], and further research is needed. The first HDI approved by FDA was vorinostat (SAHA), for the treatment of cutaneous T cell lymphoma (CTCL) in 2006 [68,69]. Three years later, in 2009, FDA has approved romidepsin, classified as a cyclic peptide, for the treatment of CTCL or/and, in 2011, peripheral T-cell lymphoma (PTCL) in patients who have received at least one prior systemic therapy [70,71]. The next drug approved by FDA was belinostat, which belongs to hydroxamic acid-type compounds, for the treatment PTCL in patients with refractory and with relapsed after prior treatment in 2014 [72]. The last approved drug was panobinostat, classified as a hydroxamic acid, for multiple melanoma treatment in 2015 [73]. To increase the effectiveness of HDIs therapy and to extend the scope of application to solid tumors, HDIs combinations with commonly used chemotherapeutic agents as well as immunotherapy are currently being tested in clinical trials, with promising results [74,75,76]. Since HDIs have proven insufficient to treat solid tumors alone, preclinical studies are ongoing to find appropriate drug combinations to make them suitable for use. Some HDIs have been successfully tested in combination in preclinical studies for many types of solid tumors, including larynx cancer [77], lung cancer [78,79], breast cancer [80], glioblastoma [81], and prostate cancer [82]. No sirtuin inhibitors have been approved by FDA so far. Preclinical studies indicate that some sirtuin inhibitors show promising in vitro results used alone against solid tumors [83,84,85,86,87]; however, the data obtained from combined-treatment research are controversial, concluding different types of pharmacological interactions between tested drugs [88,89,90,91].

## 4. SAHA and Breast Cancer

Suberoylanilide hydroxamic acid (vorinostat, SAHA; C_14_H_20_N_2_O_3_) is the first generation HDAC pan-inhibitor belonging to the hydroxamic acids group of HDIs approved by FDA [68,92,93]. The HDAC catalytic activity inhibition by SAHA is based on its binding to the zinc ion located in the enzyme catalytic domain [58,94]. It has been demonstrated that SAHA shows the anti-proliferative acitivity of human cancer cell lines [95,96,97], including BC [98,99,100].

### 4.1. SAHA Modulates Receptor Status

The presence or absence of the expression of ER, PR or HER2 receptors is critical in selecting therapy for BC patients and determines the effectiveness or failure of the applied treatment [101,102]. It has been reported that SAHA regulates essential receptors that are not normally expressed in TNBC. ERα and PR, but not HER2 receptor was re-expressed in TNBC cell lines after SAHA treatment which consequently led to the inhibition of TNBC cells growth and their sensitization to tamoxifen [103], the drug used in the treatment of ER-positive BCs (Table 2) [104,105]. On the other hand, high ERα level contributes to tumor progression and limits the effectiveness of ER-positive BCs treatment [106]. ERα expression is often associated with the resistance to hormonal therapy. Therefore, several studies indicated that depletion of ERα from BC cells could serve as a novel therapy, especially for the hormone-refractory BC [107]. It has been demonstrated that SAHA can deplete ERα at the transcriptional as well as posttranslational levels through inhibition of ERα gene expression, and stimulation of the ubiquitin-proteasome pathway degradation of ERα in MCF7 ER+ cells, respectively. SAHA-induced ERα degradation was inactivated by the heat shock protein-90 (hsp90) and enhanced by ubiquitin ligase CHIP (C-terminal Hsc70 interacting protein). Moreover, SAHA-induced ERα depletion was correlated with reduction of transcriptional activity of ERα, inhibition of cancer cell proliferation, and induction of apoptosis in MCF-7 cells (Table 2) [107].

### 4.2. SAHA Induces Cell Death and Cell Cycle Arrest

Proapoptotic activity of SAHA was investigated in luminal (MCF7 and T47D) and TBNC (MDA-MB-231) cell lines. A dose-dependent increase in the number of apoptotic cells was observed after SAHA treatment in all analyzed BC cell lines. The strongest effect was noticed in T47D ER+ cells. Moreover, FACS analysis of PI-stained cells indicated that incubation of BC cells with SAHA at relatively high concentrations (IC_50_) for 48 h led to an accumulation of cells in the G1 phase of the cell cycle corresponding with the cell reduction in the G2 phase. Similar to apoptosis, cell cycle arrest in G1 phase was more pronounced in T47D cells compared to MCF-7 and MDA-MB-231 cell lines [108]. SAHA activity was also investigated in tamoxifen-resistant MCF-7 (TAMR/MCF-7) cells. After SAHA treatment, the expression of HDAC1–4 and 7 was meaningfully reduced, whereas expression of acetylated histone 3 and histone 4 (H3Ac and H4Ac, respectively) was enhanced in TAMR/MCF7 cells. In TAMR/MCF7 cells induction of cell cycle arrest in G2/M phase in a dose-dependent manner was noticed, while on the contrary no effect was observed in MCF7 native cells. Moreover, the percentage of apoptotic cells was relatively low after SAHA treatment. However, expression of lipid phosphatidylethanolamine-conjugated form of light chain 3 II (LC3-II) and beclin-1 (autophagic cell death markers) was significantly increased in TAMR/MCF-7 cells treated with SAHA. Additionally, the in vivo study indicates that SAHA had a significant impact on tumor growth reduction, whereby no visible side effects were observed in mice bearing the TAMR/MCF-7 cell xenografts [109]. Autophagy is an important process in the degradation of the mutated form of p53 (mutp53). It has been demonstrated that SAHA significantly induced mutp53 degradation in DLD1 (mutp53-S241F) and MDA-MB-231 (mutp53-R280K) BC cell lines in context-dependent fashion. Degradation of mutp53 was correlated with induction of autophagy in MDA-MB-231 cells, while in DLD1 cells despite promoting a decrease in mutp53 level, SAHA did not increase in autophagy level (Table 2) [99].

### 4.3. SAHA Affects Migration and Epithelial-Mesenchymal Transition (EMT)

Histone acetylation plays an important regulatory role in the migration process. Cell migration stimulated by leptin, a peptide hormone secreted from adipocytes, being an independent risk factor in BC, was meaningfully inhibited after SAHA treatment in MCF7 luminal and MDA-MB-231 TNBC cells. SAHA significantly enhanced the acetylation level of H3K14 and H3K27 in MCF-7 cells, while leptin repressed these modifications [112]. Recent studies have shown that SAHA strongly affected epithelial-mesenchymal transition (EMT) in TNBC (MDA-MB-231 and BT-549) cell lines. SAHA meaningfully increased expression of E-cadherin (epithelial marker) and decreased expression of N-cadherin, vimentin, and fibronectin (mesenchymal markers of EMT), simultaneously. SAHA does not affect the nuclear translocation and expression of Twist, Snail, Slug, and ZEB EMT-related transcription factors; however, it affected forkhead box protein A1 (FOXA1) expression, a leading cancer progression factor, in MCF7 and T47D BC cells. SAHA decreased FOXA1 expression at both protein and mRNA levels, leading to downregulation of FOXA1 nuclear translocation, and ultimately caused a significantly decreased in EMT in TNBC cells. The suggested mechanism of EMT promoted by SAHA depends on the HDAC8/FOXA1 signaling pathway in TNBC cells [110]. Another research group obtained slightly divergent results. Although SAHA inhibited migration and proliferation of all analyzed BC cells in a time- and dose-dependent fashion, MDA-MB-468 cells with a more mesenchymal phenotype were found to overexpress mesenchymal markers (e.g., N-cadherin), whereas epithelial phenotype BC cells (T47D, MCF7) responded to SAHA treatment by an increase of epithelial markers (e.g., E-cadherin) expression. Therefore, induction of EMT or MET by SAHA is not a universal mechanism but cells- and context-dependent, and thus EMT should not be considered as the only measurement for tumor aggressiveness in BC (Table 2) [111].

## 5. “SAHA et al.” and Breast Cancer

Although SAHA is successfully used in CTLC therapy, the clinical trial results obtained in the monotherapy of solid tumors are not satisfactory enough [113]. Therefore, attempts are made to test the effects of SAHA in polytherapy [108,114]. There are many in vitro and in vivo studies showing the efficacy of combining SAHA with other active compounds that are currently available in the treatment of different types of cancer [92,108,114,115].

### 5.1. SAHA and Cisplatin (CDDP)

SAHA in combination with standard chemotherapeutic agent CDDP, which is mainly used in the therapy of TNBC patients [116,117,118,119], induced cell cycle progression and apoptosis as well as inhibited proliferation of T47D, MCF7, and MDA-MB-231 BC cells. Combination of CDDP and SAHA at a fixed-ratio of 1:1 determined by isobolography method exerted additive pharmacological interaction in MCF7 and MDA-MB-231 cells, and additive with a tendency towards synergism in T47D BC cells. Moreover, combination of CDDP and SAHA resulted in cell cycle arrest and an increase in percentage of apoptotic cells in all analyzed BC cell lines in comparison with a single treatment. All these findings suggest that SAHA could be combined with CDDP to optimize treatment regimens in different types of BC [108]. The influence of the Notch1 activity on the pharmacological interaction between CDDP and SAHA was also determined in MCF7 luminal [92] and TNBC cells [115]. MCF7 and MDA-MB-231 BC cells were genetically modified in order to express a differential level of Notch1 activity. It has been demonstrated that the cytotoxic effect of SAHA was higher in MCF7 cells with decreased Notch1 activity and lower in cells with increased Notch1 activity than native BC cells. In MDA-MB-231 BC cells SAHA individually or in combination with CDDP decreased expression of Notch1 gene, which overexpression is observed in patients suffering from BC. The isobolographic analysis of pharmacological interactions between SAHA and CDDP at a fixed ratio of 1:1 exerted additive interaction in MCF7 transfected cells. The combination of CDDP with SAHA in MDA-MB-231 TNBC cells with increased activity of Notch1 yielded an additive interaction, whereas additivity with a tendency towards antagonism was observed for the combination of CDDP with SAHA in MDA-MB-231 TNBC cells with decreased activity of Notch1. All these studies suggest that SAHA might be considered as a potential therapeutic agent in combination with CDDP against Notch1-altered luminal as well as some types of TNBC with altered Notch1 activity (Table 3) [92,115].

### 5.2. SAHA and Taxanes

The role of taxanes in monochemotherapy or in combination with other active agents has suggested their potential therapeutic impact on the treatment of patients with early BC. Recent studies in the adjuvant setting have shown that taxanes, used individually or in combinations, possess the capability to induce significant improvement in terms of survival of BC patients [130,131].

An immortalized human breast epithelial cell line and a panel of 8 human BC cell lines were used to examine the effect of taxol, SAHA, and their combination on inhibition of cancer cells proliferation by MTT assay. The influence of SAHA with and without taxol on apoptosis, cell cycle arrest, and protein expressions were also determined. The inhibitory effect of SAHA/taxol treatment on tumor growth was characterized in BALB/c nude mice bearing a BC xenograft in vivo. Synergistic dose-dependent growth inhibition was noticed in all analyzed BC cell lines treated with the SAHA/taxol combination. The synergetic effect of SAHA and taxol was confirmed in the xenograft cancer model in vivo. The apoptosis assay and cell cycle analysis showed that these synergistic effects resulted from enhanced apoptosis and G2/M arrest. All these findings have shown that SAHA increases the anti-tumor effect of taxol in BC both in vitro and in vivo, therefore the combination of SAHA with taxol may have therapeutic potential in the therapy of BC patients (Table 3) [120].

SAHA in combination with paclitaxel (PAX), which is commonly used in cancer chemotherapy [132], synergistically induced cell growth inhibition in taxane-resistant BC cells. Combined treatment with SAHA and PAX had a synergistic cytotoxic effect against taxane-resistant BC cells. Oligonucleotide microarray analysis identified 28 genes (ANKRD57, ATP2C1, C12orf49, DMD, EOMES, 8 ESTs, EXOC6, HCG9, IFNGR1, KIFC3, LANCL1, MAPK13, MT1G, NDRG4, NT5E, RAB4A, RGL4, SCHIP1, SMC4, SYNGR3, TM9SF3) whose expression was correlated with the combined treatment with PAX and SAHA. Twelve of these genes were down-regulated in BC cell lines that were PAX-resistant. A combination of PAX and SAHA could be an efficacious form of therapy for the treatment of BC patients, and genes involved in the synergistic response to PAX and SAHA could serve as biomarkers to predict therapeutic response in BC patients (Table 3) [121].

### 5.3. SAHA and Trastuzumab

Trastuzumab is the monoclonal antibody (mAb) used as a standard in the treatment of patients harboring HER2-overexpressing BC [133,134,135]. Antibody-dependent cell-mediated cytotoxicity (ADCC) and antibody-dependent cell-mediated phagocytosis (ADCP) are two major mechanisms of action of trastuzumab. It has been revealed that SAHA enhanced trastuzumab-independent cytotoxicity and trastuzumab-mediated ADCP. Moreover, SAHA downregulated the expression of the anti-apoptotic protein myeloid leukemia cell differentiation 1 (MCL1) and anti-phagocytic CD47 as well as induced an immunogenic cell death, characterized by the exposure of calreticulin (CALR) in SKBR3 BC cells. All these findings suggest that the immunomodulatory activity of SAHA supports a rationale combined treatment approach with trastuzumab for BC patients’ treatment (Table 3) [122].

### 5.4. SAHA and Olaparib

SAHA in combination with olaparib, a poly (ADP-ribose) polymerase (PARP) inhibitor [136,137], synergistically inhibits proliferation of a panel of 8 TNBC cell lines in vitro and in vivo in nude mice harboring TNBC xenografts. In PTEN-deficient TNBC cell lines the SAHA/olaparib synergism was weaker, while the stronger synergism was observed in BRCA1-mutated TNBC cells. Generally, TNBC cells remain resistant to PARP inhibitors in monotherapy. Treatment with SAHA can sensitize TNBC cells to olaparib in BRCA mutated as well as BRCA wild-type (wt) TNBC cells, irrespectively of their initial sensitivities to olaparib alone. It has been demonstrated that treatment of MDA-MB-157 and MDA-MB-231 with SAHA decreased the IC_50_ value for olaparib to a similar level as occurred in MDA-MB-436 BRCA1 mutated olaparib-sensitive BC cell line. In BRCA1 wt cells, the effect of the combined treatment relied on DNA damage-induced cell cycle arrest followed by induction of apoptosis. A drastic decrease in the expression of proteins involved in homologous recombination (HR), leading to a large imbalance of the ratio P-H2AX/RAD51, was observed in the HCC-1937 BRCA1-mutated cell line. All these results can provide a preclinical rationale to combine SAHA with olaparib to reduce HR efficiency in TNBC cells and sensitize these aggressive tumors to PARP inhibition (Table 3) [123].

### 5.5. SAHA and Idasanutlin (RG7388)

Idasanutlin (RG7388) is an oncogene-derived protein, being a potent mouse double minute 2 (MDM2) antagonist [138], tested in the clinical trials for the treatment of various types of carcinomas [139,140]. The latest findings revealed that SAHA in combination with RG7388 induces cell death through cell cycle arrest and cytotoxic mechanisms in MCF7 BC cells. However, the exact mechanism is still unknown. It has been demonstrated, that RG7388 treatment causes cell death by elevating p21WAF1/CIP1 through inhibition of MDM2 in LNCaP prostate cancer cells, but not in MCF-7 cells. Therefore, further studies are needed to understand the mechanism of action of combinational treatment with RG7388 and SAHA (Table 3) [140,141].

### 5.6. SAHA and Tozasertib (MK-0457)

MK-0457 (VX-680) is a small-molecule aurora kinase (AK) inhibitor [142,143] with preclinical anticancer activity [144]. MK-0457 has been assessed in phase II clinical trials in patients with Philadelphia chromosome-positive acute lymphoblastic leukemia (Ph + ALL) with the T315I mutation or treatment-refractory chronic myelogenous leukemia (CML) [145]. It has been demonstrated that SAHA increases inhibition of aurora kinases (AKs) activity, induced by MK-0457 inhibitor in MDA-MB-231 cells. AKs are overexpressed in human malignancies, and they are considered as a potential oncotarget in tumorigenesis [146]. AKs regulate multiple components of mitotic cell division in eukaryotic cells. In BC cells aurora A is frequently overexpressed or amplified leading to genomic instability, aberrant chromosome segregation, and activation of oncogenic pathways. Therefore, the effect of co-treatment of SAHA and MK-0457 in MDA-MB-468, MDA-MB-231 TNBC and BT-474 BC cells exhibited aurora A amplification were investigated. Treatment with MK-0457 depleted p-AKs (phosphorylated-AKs) level and their activity as well as induced cell cycle arrest in G2/M phases, multipolar mitotic spindles, DNA endoreduplication, and apoptosis of the BC cells. A similar effect was observed with the MLN8237 aurora A-specific inhibitor treatment. Treatment with SAHA induced hsp90 acetylation and inhibited its chaperone association with AKs, leading to depletion of AKs and survivin. Exposure of the siRNA to AK A also induced apoptosis, which was augmented by co-treatment with MK-0457 and SAHA. Interestingly, co-treatment with SAHA enhanced MK-0457-mediated inhibition of the aurora A and aurora B activities, leading to synergistic in vitro activity against human BC cells. Moreover, treatment with MK-0457 and SAHA in combination caused greater inhibition of tumor growth as well as superior survival of mice bearing MDA-MB-231 xenografts. All these findings suggest that combined treatment with MK-0457 and SAHA could be a novel promising therapeutic strategy for the treatment of aurora A-amplified and/or TNBC (Table 3) [124].

### 5.7. SAHA and Epigallocatechin-3-Gallate (EGCG)

SAHA individually or in combination with epigallocatechin-3-gallate (EGCG), a DNA methyltransferase (DNMT) inhibitor isolated from green tea [147,148], was administered to TNBC cells in vitro. SAHA and EGCG reduced the metastatic potential of TNBC by inhibiting migration of TNBC cells across a fibronectin matrix and inducing the apoptotic pathway. Drugs in combination increased the expression of pro-apoptotic caspase 7 and decreased the expression of cellular inhibitor of apoptosis 2 (cIAP2). cIAP2 degradates caspases by linking them to ubiquitin molecules. The expression of cIAP2 is upregulated in TNBC and plays a role in the EMT (Table 3) [125].

### 5.8. SAHA and Sodium Butyrate (NaB)

The combination of SAHA with another HDI, sodium butyrate (NaB) [149,150] has a strong synergistic effect on inhibition of cell proliferation, cell cycle arrest in G0/G1 phase, and promotion of apoptosis in TNBC cells. Moreover, both inhibitors downregulated phosphorylation of mutant p53, as well as its protein and mRNA expression level, wherein a similar inhibition effect was not observed for wild-type p53 in TNBC cells. It was demonstrated that SAHA reduces the binding of Yin Yang 1 (YY1) transcription factor with human p53 promoter in the -96 to -102 position. Further, SAHA inhibits the association of YY1 and HDAC8, increases acetylation of residues 170–200 in YY1, and as a consequence decreases its transcription activities, and finally suppresses YY1 induced p53 transcription. Summarizing, HDAC8/YY1/mtp53 signals act as an important target for TNBC; therefore, SAHA can be considered as a drug candidate for the treatment of TNBC (Table 3) [126].

### 5.9. SAHA and Clarithromycin (CAM) + Bortezomib (BZ)

SAHA in combination with clarithromycin (CAM), the 6-O-methyl ether of erythromycin A being a macrolide antibiotic used in the treatment of respiratory tract, skin and soft-tissue infections [151,152], and bortezomib (BZ) an antineoplastic agent that is used in the treatment of refractory multiple myeloma and certain lymphomas [153,154], enhances ER stress-mediated cell death through concomitant targeting of aggresome formation and intracellular proteolytic pathways in MDA-MB-231 BC cells. Combined treatment of CAM, BZ and SAHA enhanced the apoptosis-inducing effect compared with treatment using each drug alone or a combination of two. Moreover, expression levels of ER-stress-related genes, including CHOP and GADD153, the pro-apoptotic transcription factors, were induced after simultaneous treatment of three active agents. CHOP protein undergoes phosphorylation by the p38 MAP kinase family, which enhances its transcriptional ability for different pro-apoptotic genes, including BAX, BIM, DR5 or TRB3 (Table 3) [127].

### 5.10. SAHA and Tumor Necrosis FACTOR Related Apoptosis Inducing Ligand (TRAIL)

The in vivo research showed that SAHA is able to sensitize tumor necrosis factor related apoptosis inducing ligand (TRAIL)-resistant BC cells. BALB/c nude mice were orthotopically implanted with MDA-MB-468 TRAIL-resistant cells, and then treated with TRAIL, SAHA or SAHA followed by TRAIL 4 times for 3 weeks. It has been demonstrated that SAHA decreased MDA-MB-468 xenografts growth via inhibition proliferation, angiogenesis and metastasis markers as well as through cell cycle arrest and induction of apoptosis. Additionally, SAHA downregulated expression of nuclear factor-kappa B (NF-κβ), which is a transcription factor contributing to the malignant phenotype, and different proteins related to NF-κβ (Bcl-xL, Bcl-2, cyclin D1, vascular endothelial growth factor (VEGF), hypoxia-inducible factor-1-alpha (HIF1α), interleukin-6 (IL-6), interleukin-8 (IL-8), matrix metalloproteinase-2 (MMP-2), and matrix metalloproteinase-9 (MMP-9)). In turn, proapoptotic proteins such as Bax, Bim, Noxa, p21/Cip1 and PUMA, as well as DR4, DR5, tissue inhibitor of metalloproteinase-1 (TIMP1) and tissue inhibitor of metalloproteinase-2 (TIMP) were upregulated in BC cells after SAHA treatment. Interestingly, the sequential treatment of nude mice with SAHA followed by TRAIL was much more effective in inhibiting tumor growth, angiogenesis and metastasis, as well as inducing apoptosis than SAHA alone. Moreover, mice from the control group had increased numbers of von Willebrand factor-positive blood vessels and CD31(+) as well as increased circulating vascular endothelial growth factor receptor 2-positive endothelial cells compared with SAHA-treated or SAHA plus TRAIL-treated mice. Summarizing, sequential treatment with SAHA followed by TRAIL targets multiple pathways in tumor progression, angiogenesis and metastasis, and can represent a novel therapeutic approach in the BC treatment (Table 3) [128].

### 5.11. SAHA and Soluble CD137 Receptor

SAHA upregulates CD137 protein expression, which belongs to the tumor necrosis factor (TNF) superfamily, in MDA-MB-231 BC cells. Moreover, SAHA reinforced destruction of BC cells caused by soluble CD137 receptor itself. Upregulation of the CD137 receptor/ligand system correlates with a synergistic cytotoxic effect of combined treatment with SAHA and soluble CD137 receptor in MDA-MB-231 cells. All these findings suggest that the combination of SAHA with TNF-related receptor could be a new therapeutic approach for the treatment of BC patients (Table 3) [129].

## 6. SAHA in Clinical Trials

The potential of SAHA individually or in combination with other anticancer drugs is under evaluation in numerous clinical trials (Table 4) [155,156,157,158,159,160,161,162,163,164,165,166,167,168,169,170,171,172,173,174].

In relapsed or refractory breast, colorectal, or non-small cell lung cancer (NSCLC) SAHA was administered in an early phase II clinical trial. A cohort of 16 patients was enrolled in this study including 3 with BC, 3 with lung cancer and 10 with NSCLC. The dosing regimen of oral SAHA was 400, 300, or 200 mg twice daily for 14 consecutive days followed by a 7-day rest. Due to the heavy toxicities events reported and as no patients were observed to have a partial response (PR) or complete response (CR) according to the RECIST (Response Evaluation Criteria In Solid Tumors) the study was terminated early. Yet it was found that neither the total daily dose: 600 or 800 mg of oral SAHA for 14 days with a 7-day rest was tolerable [113].

A lower dose of SAHA in monotherapy was administered in a phase II trial in patients with metastatic breast cancer (MBC). Among 14 women with measurable MBC 6 patients (43%) were ER/PR negative and 10 (71%) were HER-2 negative at diagnosis. The patients received SAHA at a dose of 200 mg orally twice daily, for the first 14 days of each 21 day cycle. The drug was well tolerated, yet the observed responses were not adequate for RECIST single-agent activity response criteria as stable disease (SD) was observed in 4 (29%) patients with a median progression-free survival of 8.5 months (range 4–14 months). The median progression-free survival was 2.6 months while the median overall survival was 24 months for the 14 patients and overall survival at 12 months was 71%. Nonetheless, the study showed that SAHA in monotherapy induces tolerable toxicities together with modest clinical benefit in terms of stable disease, giving a basis for future studies including SAHA in combination with other agents [175].

Additionally, the effect of short-term SAHA administration in women with primary BC prior to definitive breast surgery or other primary treatment was studied; however, the aim of the study was an evaluation of several biomarkers associated with cancer. The group of 25 patients received the treatment (300 mg of SAHA given twice a day for 3 out of 7 days), while 29 patients had not and followed standard pre-surgery procedures (untreated control). Several weak toxicities were reported due to the treatment. Pre- and post-SAHA treatment biopsy samples were evaluated for candidate biomarkers that may predict response to the drug. Though, the number of evaluable for prespecified marker analysis samples was lower than expected the data showed statistically significant greater reductions in the mRNA expression of the proliferation-associated genes: Ki-67, STK15 and cyclin B1, and non-statistically significant greater reduction of MYBL2 and survivin genes, in the samples from SAHA-treated women as compared to control samples. The additional analysis of expression of Ki-67 or cleaved caspase-3 by immunohistochemistry did not confirm such a trend. Also, methylation of candidate genes changes were not observed [176].

The ability to metabolize SAHA in order to predict its clinical outcomes in Asian women with BC was tested. A key enzyme involved in SAHA metabolism is UDP-glucuronosyltransferase 2B17 (UGT2B17), a gene of which deletion variant is common in the Asian population. The in vitro studies have shown UGT2B17 reduced enzymatic activity in UGT2B17 null genotype (UGT2B17*2). For SAHA monotherapy women with advanced anthracycline and taxane pretreated BC were enrolled and genotyped for UGT2B17*2. In the I–II phase study, the cohort of twenty-six patients was receiving 400 mg of SAHA daily continuously in 21-day cycles (range 1–10). The trial was completed with no CR, one PR and six SD lasting for 12 weeks or more, and 19 progressive disease (PD). The UGT2B17*2 homozygotes (representing 62% of patients) glucuronidated SAHA less efficiently by approximately 30% compared with those with at least one wild-type allele, and trended toward having higher SAHA efficacy and toxicity. Consistently, more clinical benefit and longer progression-free survival (PFS) with SAHA treatment were reported for UGT2B17*2 null genotype [176]. These findings suggest that differences in SAHA pharmacodynamics may be linked with genotype differences.

Promising results from certain clinical trials that employed SAHA in combination with other chemotherapeutic drugs were reported. The phase I trial was performed to analyze a combination of SAHA and DOX in solid tumors mostly melanoma, BC, lung cancer, sarcoma, colon cancer and several others. Among 32 enrolled patients, five patients with BC (16%) were treated. SAHA administered at 400, 600, 800, or 1000 mg daily on days 1–3, followed by DOX (20 mg/m^2^) on day 3 for 3 of 4 weeks. The authors founded that SAHA administration for 3 days before DOX therapy for 3 of 4 weeks, increases the maximum tolerated dose of SAHA to 800 mg per day (400 mg twice daily for five doses) in patients with advanced solid tumor malignancies. Finally, antitumor activity in 24 evaluable patients included two PR (8%; breast and prostate cancer) and SD for more than 8 months in two patients (melanoma). Correlative studies have shown histone H3 and H4 hyperacetylation changes in peripheral blood mononuclear cells (PBMCs) and tumor cells at a comparable level. Histone hyperacetylation seemed to correlate with HDAC2 expression at a baseline. Also, SAHA effects on downstream targets associated with chromatin decondensation, such as depletion of heterochromatin protein 1 (HP-1) and DNA topoisomerase IIα expression, were observed in 66% of PBMCs samples [177].

Similarly, SAHA can be safely combined with PAX and bevacizumab in metastatic BC as shown in the phase I–II study [178]. To overcome the potential cumulative toxicity associated with continuous or more protracted drug schedules the intermittent SAHA administration was performed. The treatment of cohort of fifty-four patients with measurable disease and no prior chemotherapy for MBC was SAHA (200 or 300 mg twice daily) on days 1–3, 8–10, and 15–17, plus PAX (90 mg/m^2^) on days 2, 9, 16 and bevacizumab (10 mg/kg) on days 2 and 16 every 28 days. PAX was administered before bevacizumab and prior to PAX standard medications including dexamethasone were provided. As a result, 49% of treated and eligible patients exhibited an objective response and 30% of patients had stable disease for ≥24 weeks. The observed responses were similar between patients with ER-positive and ER-negative BC (50% vs. 44%). Moreover, the evaluation of paired tumor and blood samples before and after the third SAHA dose prior to administration of other antineoplastic therapy revealed the increased acetylation of alpha tubulin and the K69 (lysine 69) residue of hsp90, induction of the stress protein hsp70, induction of p27 and p21 expression, and downregulation of cyclin-dependent kinase (CDK) 4, confirming the effects of SAHA reported in preclinical studies [179,180].

The combination of SAHA with albumin-bound PAX and other cytotoxic agent carboplatin was also under evaluation in women with HER2-negative BC [181]. In the II phase clinical trial the patient cohort (62 women) received 12 weeks of preoperative cytostatics carboplatin (AUC 2 weekly) and nab-PAX (100 mg/m^2^ weekly) with SAHA (400 mg oral daily, days 1–3 of every 7 day period) or placebo. After 15 days since the first treatment with SAHA/placebo and prior to receiving carboplatin and nab-PAX, the patient-derivative samples were tested to assess the DNA methylation status for BC [182,183]. The calculated cumulative methylation index (CMI) was established for ten selected genes including HIST1H3C, AKR1B1, GPX7, HOXB4, TMEFF2, RASGRF2, COL6A2, ARHGEF7, TM6SF1, and RASSF1A, which were unmethylated or methylated at low levels in normal breast tissue, but frequently highly methylated in BC tumors of all stages. The obtained results showed that a high DNA methylation level may predict poor patients’ response. However, the usage of DNA methylation status as a predictive marker for BC outcome and response to systemic therapy needs further larger prospective studies [184].

Due to preclinical studies, HDIs can reverse the ER modulators such as tamoxifen and aromatase inhibitor (AI) resistance in hormone receptor-positive BC [185,186]. The clinical study evaluating the combination of SAHA with tamoxifen was carried out. The 43 pre- and post-menopausal women with ER- or PR-positive MBC without ovarian suppression in conjunction with AI treatment were enrolled. The additional patients’ requirements were (1) progression in any number of AI for metastatic disease or (2) recurrence of disease during adjuvant AI or (3) completed tamoxifen therapy for at least 1 year by pre-menopausal women. SAHA was given at a dose of 400 mg once daily for 21 of 28 days and tamoxifen at a dose of 20 mg daily without interruption, what allowed to the distinction between SAHA toxicities alone and toxicities associated with both of the drugs. Due to the toxicities observed in around 30% of patients SAHA dose was reduced to 300 mg, obtaining satisfactory drug mixture tolerance. The obtained results were quite promising as the confirmed objective responses by RECIST criteria were seen in 19% of patients and stable disease for more than 24 weeks in 21% of patients and the median time to progression was 10.3 months. Additionally, changes in histone H4 acetylation were measured in PBMCs in pre- and post-treatment (day 8) samples and showed statistically more pronounced histone H4 acetylation in patients with a response or stable disease. Also, higher baseline expression of HDAC2 in PBMCs was associated with a more a pronounced increase in histone H4 acetylation. These results confirmed the use of HDAC2 expression as a predictive marker and histone hyperacetylation marks as pharmacodynamic markers for the efficacy of the SAHA and tamoxifen combination [187]. Similarly, to potentially overcome AI resistance in patients whose tumors may have endocrine sensitivity SAHA was administered sequentially or simultaneously with AI [188]. The 8 women of the sequential cohort were given SAHA, 400 mg orally daily for 2 weeks, followed by an AI daily for 6 weeks, while 15 patients enrolled in the simultaneous cohort was given the same dose of SAHA daily concomitantly with the AI for 5 consecutive days in 3 weeks, with the fourth week off, in two 28-days cycles. The endocrine sensitivity was monitored by 18F-fluoroestradiol positron emission tomography (PET) measuring ER status and 18F- fluorodeoxyglucose (FDG) PET scans showing tumor glycolytic activity. The scans were performed at baseline, week 2, and week 8. Also, conventional imaging (CT, bone scanning) was performed at baseline and week 8, and patients were followed for PFS. The positive outcome was reported for 8 patients who had SD at week 8, and in 6 of these 8 patients had SD for more than 6 months. Two patients had benefits reaching 16 and 21 months until progression. In this study SAHA exposure did not increase the 18F-fluoroestradiol uptake, suggesting that SAHA treatment does not change the binding affinity for estradiol. Yet, the higher baseline 18F-fluoroestradiol uptake was associated with longer PFS [189,190].

Due to the poor prognosis for another hormone receptor-HER2 positive BC the new therapies targeting HER-2 are under evaluation, e.g., treatment with the monoclonal antibody trastuzumab (herceptin) directed against the extracellular domain IV of HER2 [191]. One of the mechanisms of trastuzumab activity is the inhibition of the MAPK and PI3K/Akt pathways, which leads to an increase in cell cycle arrest, and the suppression of cell growth and proliferation [192]. Although the reported benefits of the trastuzumab therapy in HER-2 overexpressing BCs [193,194], the trastuzumab resistance occurs quite often [195,196]. As SAHA attenuates the levels of pAKT and c-RAF-1, its combination with trastuzumab was tested in phase I/II study to overcome trastuzumab resistance in patients with HER2 overexpressing BCs [197]. SAHA was administered at 200 mg twice daily on days 1–14 combined with 6 mg/kg trastuzumab on day 1 every 21 days. The 16 patients mostly with confirmed HER2+ BC and reported progressive disease after prior treatment with trastuzumab was treated with SAHA, without any satisfactory response. Therefore, the study was terminated due to the insufficient statistical evidence that the addition of SAHA reverses trastuzumab resistance in these patients. Nonetheless, in clinical trials utilizing combination of SAHA with other components trastuzumab was used additionally for HER2/neu positive disease. For example, in a phase I–II study SAHA was administered with PAX (and trastuzumab) followed by doxorubicin-cyclophosphamide (AC) in patients with operable or locally advanced BC who were candidates for neoadjuvant chemotherapy. The 55 patients enrolled for the II phase of the trial were divided into three groups: (A) HER2+ (26 patients), (B) ER/PR/HER2- (16), (C) ER+ and/or PR+ and HER2- (13). The treatment with PAX and trastuzumab weekly (12 consecutive week) plus SAHA (300 mg PO twice a day on days 1—3 of each PAXf/trastuzumab dose), followed sequentially by dose-dense doxorubicin-cyclophosphamide for 4 cycles, was found to be well-tolerated and produced breast and nodal pCR in 24%, 54% and 0% patients of the A, B and C stratum, respectively. The in vivo data from untreated and post-SAHA treated tumor biopsy samples showed that SAHA increased acetylation of hsp90 and alpha tubulin, and reduced expression of HDAC6 and hsp90, leading to depletion of hsp90 client proteins that promote cell survival. These results strongly support further evaluation of SAHA with cytotoxic and HER2/neu-directed therapies in BC. The expression analysis of several biomarkers (such as HDAC6, p21, p27, and Ki67) did not reveal their association with pathologic response to therapy [197].

Regarding growing evidence that HDIs and proteasome inhibitors may act synergistically in malignancies the combination of SAHA and bortezomib in patients with advanced solid tumors including breast tumors, was conducted. The cohort of 29 patients was enrolled: 13 men and 16 women suffering from several cancer types such as sarcoma, pancreatic, colorectal, gastrointestinal stromal tumor (GIST), and breast (2 patients). The majority of patients were pretreated, having received 2 or more prior chemotherapy regimens. The MTD (maximum tolarated dose) was established at SAHA 300 mg twice a day on days 1–4 and 8–11 and bortezomib 1.3 mg/m^2^ IV on days 1, 4, 8 and 11 of a 21 day cycle. Multiple patients with treatment-refractory cancer achieved stable disease with this dosing regimen, especially in heavily pre-treated sarcoma, colorectal carcinoma, and GIST. The trial included only two patients with breast tumors, yet overall obtained data are promising [198].

## 7. Chimeric HDAC-Based Inhibitors

An emerging class of HDAC-based inhibitors with improved affinity and efficacy in the treatment of relapsing and drug-resistant cancers has gained attention recently. Molecules called chimeric HDAC-based inhibitors are combined of two pharmacophores, one comes from HDAC inhibitor, the second from another anticancer drug, connected via an inert linker which undergoes enzymatic cleavage in vivo. In order to increase the potency of anticancer agents, the hydroxamic acid chelating group in SAHA was combined with the active groups of different compounds such as receptor and non-receptor tyrosine kinase inhibitors, topoisomerase inhibitors, DNA damaging agents, nuclear receptors targeting agents, androgen/estrogen receptors inhibitors, vitamin D receptors agonist and others. Both parent compounds act on different targets and therefore the chimeric inhibitors are able to simultaneously regulate multiple pathways. Importantly, the chimeric drugs mostly do not cause drug resistance or additive toxicity often observed in the combination therapy [199,200]. One of the developed anticancer hybridized HDAC inhibitor is CUDC-101 composed of hydroxamic acid linked with the quinazoline core of erlotinib acting as a receptor (EGFR and HER2) tyrosine kinase inhibitor [201]. CUDC-101 exhibited potent antiproliferative and proapoptotic activity in lapatinib-sensitive (HER2 positive) and resistant (HER2 negative) BC models. Mechanistic studies have shown that CUDC-101 simultaneously inhibited HDAC, EGFR, and HER2 expression but also indirectly attenuated signaling mediated by HER3, MET (mesenchymal-epithelial transition), AXL, and AKT [202,203]. Due to its great potential to overcome cancer resistance and tumor metastasis CUDC-101 given intravenously was evaluated in phase I clinical trial in patients with advanced solid tumors (including 6 patients with BC) with promising results [204].

## 8. SAHA and Drug Carriers

Combined chemotherapy and nanomaterials (NMs)-drug delivery system are two areas that have shown significant promise in the therapy of cancer patients. Conjugation of nanoparticles with anti-tumor drugs allows to control the drug release and reduce the toxicity of active agents. Erlotinib is the inhibitor of epidermal growth factor receptor (EGFR), which is highly over-expressed in many types of solid tumors, e.g., breast, ovarian, lung, colorectal, or head and neck cancers. Increased activity of EGFR leads to drug resistance and consequently poor prognosis. Erlotinib is highly selective for the tyrosine kinase, resulting in inhibition of proliferation, induction of apoptosis, and cell cycle arrest in cancer cells. SAHA and erlotinib (ERL) loaded titanium oxide IV (TiO_2_) nanoparticles (NPs) were used for the treatment of MDA-MB-231 TNBC and MCF-7 luminal BC cells and human cancerous amniotic cells (WISH). Cell viability has been significantly decreased and the number of apoptotic cells has been increased when both types of BC cells were treated with ERL, SAHA, or dual drug-loaded TiO_2_ NPs compared with bare TiO_2_ NPs treatment and control cells. Moreover, ERL and SAHA-loaded TiO_2_ NPs treatments arrested BC cells at the G2/M phase. It has been demonstrated that partner and localizer of BRCA2 (PLAB2) gene expression was upregulated in ERL and SAHA-loaded TiO_2_ NPs compared with control cells. Summarizing TiO_2_ NPs can be used as a nanocarrier for chemotherapy drugs like ERL or SAHA [100].

PEG-hydrophobic-based drug conjugates (pro-drugs) are promising nanocarriers that have the function of delivery as well as intrinsic anti-tumor activity. The effect of SAHA on reactivation of the ERα expression and synergism with TAM, a selective estrogen-receptor modulator, were investigated. Moreover, SAHA prodrug-based dual-functional nanocarrier was developed for co-delivery of SAHA and TAM for effective combined therapy of BC. Both, SAHA and SAHA-containing polymeric nanocarrier (POEG-co-PVDSAHA) were induced the re-expression of ERα in TNBC cells, which may help in sensitization of TNBCs to TAM. Interestingly, POEG-co-PVDSAHA self-assembled to form small-sized micellar carriers, effective in formulation and co-delivery of TAM. TAM-loaded POEG-co-PVDSAHA micelles demonstrated enhanced synergistic cytotoxic effect against TNBC cells compared with free TAM, free SAHA, and TAM loaded into POEG-co-PVMA, a pharmacologically inert control carrier. It has been demonstrated that delivery of TAM via POEG-co-PVDSAHA micelles leads to significant improvement of anti-tumor efficacy in 4T1.2 tumor model compared with TAM-loaded POEG-co-PVMA micelles and combination of free SAHA and TAM. SAHA prodrug-based dual-functional nanocarrier co-delivery system may provide a simple and effective strategy to re-sensitize TNBC cells to TAM-based hormone therapy [205].

PAX was bound to SAHA to form co-prodrugs based on the synergistic anticancer effects of these active agents. The PAX-SAHA co-prodrugs were conjugated by succinic acid and glycine to form the co-molecule which has shown better activity in cytotoxicity, cell cycle arrest, and tumor-suppression. Therefore, PAX-SAHA-glycine was prepared then to nanomicelles with mPEG2000-PLA1750 as the carrier using the thin film method. PAX-SAHA co-prodrug spherical nanomicelles with a particle size of 20–100 nm. It has been demonstrated in in vitro drug release test that PAX-SAHA-glycine nanomicelles had a sustained release effect, which can reduce the resistance of PAX. Cytotoxicity of combinations was evaluated by SRB assay in MCF-7, HCT-116, and drug-resistant MCF-7/ADR BC cells. The results showed that PAX-SAHA-glycine nanomicelles had better cytotoxicity than PAX, especially against the MCF-7/ADR BC cells. All the studies suggest that PAX-SAHA co-prodrug nanomicelles are a promising form of treatment in patients harboring PAX resistance BC [206].

## 9. Discussion

BC is one of the leading causes of cancer-related morbidity and mortality among women worldwide [207]. The idea of treating BC patients with new active agents capable to re-establish expression of tumor suppressor genes, which are silenced by epigenetic mechanisms is being tested [67]. HDIs seem to be a promising group of anticancer drugs, particularly in combination with other anticancer agents [84,208] or radiotherapy [209,210]. So far, 4 HDIs have been approved by the US FDA for the treatment of certain types of hematological malignancy [67]. Even though the effect against hematological cancers is rewarding, HDIs monotherapy turned out not to be compelling enough for solid tumors. Therefore, many other HDIs are being tested in clinical trials for the therapy of both hematological and solid malignancies in combination with other active agents [58]. However, some of them seem to give sufficient patient outcomes, so there is a high need to investigate their mechanism of action alone and in combination. SAHA (Zolinza^®^) can be used as a good example of effective HDI, as it was the first HDIs approved by the US FDA in 2006 for the treatment of CTCL in patients who have progressive, persistent or recurrent disease or following two systemic therapies [69]. SAHA is a moderately orally bioavailable inhibitor of HDACs classes I and II [211]. Preliminary evidence of anticancer activity of SAHA, in monotherapy or in combination with other systemic therapies, has been observed across a range of malignancies. Numerous pre-clinical studies of SAHA in combination with other anticancer agents (e.g., CDDP, taxol, trastuzumab, olaparib) or HDI (NaB) have demonstrated synergistic or additive pharmacological interactions in different subtypes of BC cells, which is the promising basis for clinical trials. Combined chemotherapy or radiochemotherapy are frequently used in preference to single-agent therapy to maximize treatment efficacy, but can be limited with increased toxicity. SAHA, as an HDI, has a different mechanism of action compared to many other anticancer drugs, therefore, it can improve clinical efficacy in combination with other systemic agents without overlapping toxicities. Moreover, increasingly sophisticated drug delivery methods such as nanocarriers can reduce drug administration dosage, ultimately reducing the toxicity of targeted therapy, what is critical for SAHA implementation. Results of clinical trials from phases I and II support the rationale for combining SAHA with other chemotherapeutic agents in order to increase the therapeutic index of anticancer regimens. Moreover, analysis of combined safety data demonstrates that SAHA has acceptable safety and tolerability profiles either in monotherapy or in combination in patients with a variety of types of BCs [211]. On the other hand, although SAHA has been market as an oral pill, its bioavailability is relatively low. The apparent half-life of oral SAHA ranged from 91 to 127 minutes [212]. A lot of clinical trials failed simply because of this factor and therapeutic biomarkers were never check. Moreover, the limited aqueous solubility of SAHA may also affect its oral bioavailability. Therefore, it is of interest to develop new formulations of SAHA for both oral and parenteral use [213].

## 10. Conclusions

**Figure 2:**
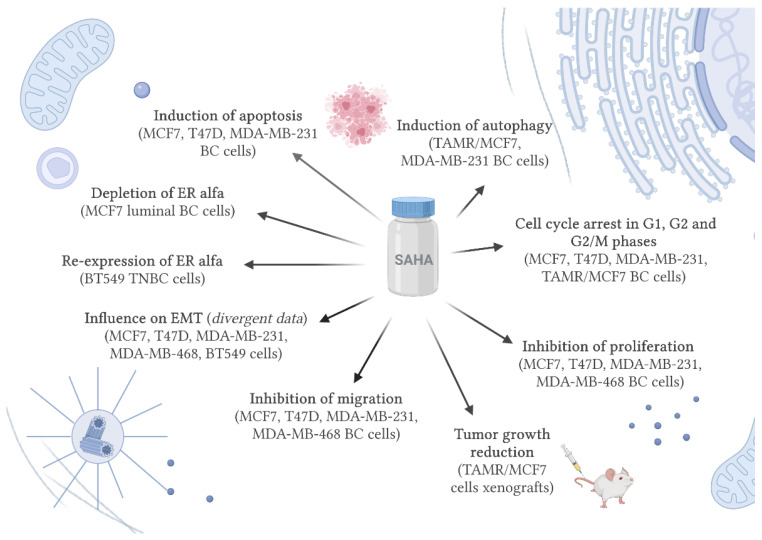
The functional mechanism of vorinostat (SAHA) in breast cancer in vitro and in vivo settings (BC-breast cancer, ER-estrogen receptor, EMT-epithelial-mesenchymal transition, SAHA-vorinostat, TAMR-tamoxifen-resistant, TNBC-triple-negative breast cancer).

SAHA, an inhibitor of class I and II of HDACs, is an effective anticancer agent which, individually and/or in combination with other conventional chemotherapeutics, exhibit anti-neoplastic properties through inhibition of proliferation, migration and invasion, induction of differentiation and apoptosis as well as cell-cycle arrest, in many types of BC cells, both in in vitro and in vivo settings (Figure 2) [214].

Interestingly, data from clinical trials show that SAHA can be well tolerated and demonstrates limited toxicity, which is rapidly reversible upon discontinuation of the drug [215]. An additional advantage of SAHA is its ability to cross the blood–brain barrier preventing the formation of brain metastases [115,216]. SAHA, alone or in combination strategies, show promising activity in BC and could have implications for the future targeted treatment of BC patients. However, further investigations are needed to evaluate the efficacy, and to provide optimal treatment regimens.

## Figures and Tables

**Table 1:** Molecular subtypes of breast cancer (BC—breast cancer, ER—estrogen receptor, PR—progesterone receptor, HER2—human epidermal growth factor receptor, Ki67—proliferation index marker, TNBC—triple negative breast cancer) [6,7,8,9].

Subtype of BC	Immunohistochemistry Status	Grade	Prevalence	Outcome	Treatment
Luminal A	ER+, PR+, HER2−, Ki67−	1/2	23.7%	good	endocrine therapy alone or with chemotherapy
Luminal B	ER+, PR+, HER2−, Ki67+	2/3	38.8%	middle	endocrine therapy and chemotherapy without/with anti-HER2 therapy
	ER+, PR+, HER2+, Ki67+		14%	poor	
HER2-overexpressed	ER−, PR−, HER2+	2/3	11.2%	poor	chemotherapy and anti-HER2 therapy
TNBC	ER−, PR−, HER2−, basal marker+	3	12.3%	poor	chemotherapy
Normal-like	ER+, PR+, HER2−, Ki67−	1/2/3	7.8%	middle	endocrine therapy alone or with chemotherapy

**Table 2:** Mechanism of action of vorinostat (SAHA) in in vitro and in vivo breast cancer (BC) pre-clinical setting (BC—breast cancer, EMT—epithelial mesenchymal-transition, ER—estrogen receptor, FOXA1—forkhead box A1, LC3-II—microtubule-associated protein 1A/1B light chain 3-II, mutp53—mutated p53, TAMR—tamoxifen resistant, TNBC—triple-negative breast cancer).

Cellular Process	Subtype of BC	Cell Line	Mechanism of Action	References
**Apoptosis**	Luminal	MCF7, T47D	-increase in the number of apoptotic cells	[108]
	TNBC	MDA-MB-231		
**Autophagy**	Luminal	TAMR/MCF7	-increase in expression of autophagic cell death markers (LC-II and beclin-1)	[109]
	TNBC	MDA-MB-231	-induction of autophagy	[99]
**Cell cycle**	Luminal	MCF7, T47D	-accumulation and reduction of cells in G1 and G2 phases, respectively	[108]
	TNBC	MDA-MB-231		
	Luminal	TAMR/MCF7	-cell cycle arrest in G2/M phases	[109]
**Proliferation**	Lumianal	T47D, MCF7	-inhibition of proliferation	[110]
	TNBC	MDA-MB-231, MDA-MB-468		
**Tumor growth**	Luminal	TAMR/MCF7 cells xenografts	-tumor growth reduction	[109]
**Migration**	Luminal	MCF7	-inhibition of migration stimulated by leptin	[111]
	TNBC	MDA-MB-231, MDA-MB-468		
	Luminal	T47D, MCF7	-inhibition of migration	[110]
	TNBC	MDA-MB-231, MDA-MB-468		
**EMT**	TNBC	MDA-MB-231, BT549	-increase in E-cadherin expression, -decrease in N-cadherin, vimentin and fibronectin expression, -inhibition of EMT through downregulation of FOXA1 expression at both mRNA and protein levels	[112]
	Luminal	T47D, MCF7	-increase in E-cadherin expression	[110]
	TNBC	MDA-MB-468	-increase in N-cadherin expression	
**ER receptor status**	Luminal	MCF7	-depletion of ERα, -inhibition of ERα mRNA, -ubiquitin-proteasome pathway degradation of ERα (inhibition of cell proliferation, induction of apoptosis)	[107]
	TNBC	BT549	-re-expression of ERα (inhibition of cell growth and sensitization to tamoxifen)	[103]

**Table 3:** Mechanism of action of vorinostat (SAHA) and other anticancer drugs in combination in in vitro and in vivo breast cancer (BC) pre-clinical settings (BC- breast cancer, BZ- bortezomib, CAM- clarithromycin, CDDP- cisplatin, DNA- deoxyribonucleic acid, EGCG- epigallocatechin-3-gallate, ER- endoplasmic reticulum, HER2-human epidermal growth factor receptor 2, MK-0457- tozasertib, NaB- sodium butyrate, PAX- paclitxel, TNBC- triple negative breast cancer, TRAIL- tumor necrosis factor related apoptosis inducing ligand).

Drug-Drug Combination	BC-Subtype	In Vitro/In Vivo Model	Mechanism of Action	Type of Pharmacological Interaction	References
SAHA and cisplatin (CDDP)	Luminal	MCF7, T47D cells	-cell cycyle progression, -induction of apoptosis, -inhibition of proliferation	-additive (MCF7), -additive with a tendency towards synergism (T47D)	[108]
	TNBC	MDA-MB-231 cells	-cell cycyle progression, -induction of apoptosis, -inhibition of proliferation, -decrease in *Notch1* expression	additive	[108]
	Luminal	MCF7 cells with increased and decreased Notch1 activity	-inhibition of proliferation	additive	[92]
	TNBC	MDA-MB-231 cells with increased and decreased Notch1 activity	-inhibition of proliferation	-additive (MDA-MB-231 with increased Notch1 activity), -additive with a tendency towards antagonism (MDA-MB-231 with decreased Notch1 activity)	[115]
SAHA and taxol	Luminal	MCF7, MCF7/ADR, T47D cells	-induction of apoptosis, -cell-cycle arrest in G2/M phases, -cell growth inhibition	synergistic	[120]
	TNBC	MDA-MB-231, MDA-MB-453, BT474 cells			
	HER2-overexpressed	SKBR3 cells			
	TNBC	BALB/c mice bearing a BC xenografts	-tumor growth inhibition		
SAHA and paclitaxel (PAX)	Luminal	MCF7, T47D, YCC-B1, YCC-B3, YCC-B5 cells	-upregulation of *MAPK13, ATP2C1, ANKDR57, MT1G, RGCR, C12orf49, EXOC6 RAB4A, TM9SF3, IFNGR1* gene expression, -downregulation of *DMD, HCG9, KIFC3, SYNGR3, NDRG4, NT5E, EOMES, SMC4, LANCL1, SCHIP1, 2EST* gene expression	synergistic	[121]
	HER2-overexpressed, TNBC	SKBR3, MDA-MB-231, YCC-B2 cells		antagonistic	
SAHA and trastuzumab	HER2-overexpressed	SKBR3 cells	-enhancement of trastuzumab-dependent cytotoxicity and phagocytosis	synergistic	[122]
SAHA and olaparib	TNBC	MDA-MB-157, MDA-MB-231, MDA-MB-436 cells	-inhibition of proliferation, -DNA-demage induced cell cycle arrest, -induction of apoptosis	synergistic	[123]
SAHA and tozasertib (MK-0457)	TNBC	MDA-MB-231, MDA-MB-468, MDA-MB-474 cells	-induction of apoptosis, -cell cycle arrest in G2/M phases, -induction of multipolar mitotic spindles, -induction of DNA endoreduplication	synergistic	[124]
	TNBC	mice bearing MDA-MB-231 xenografts	-tumor growth inhibition		
SAHA and epigallocatechin-3-gallate (EGCG)	TNBC	MDA-MB-157, MDA-MB-231	-inhibition of migration, -induction of apoptosis, -increase in *caspase-7* and *cIAP2* gene expression	synergistic	[125]
SAHA and sodium butyrate (NaB)	TNBC	MDA-MB-231, BT-549	-inhibition of cell proliferation, -cell cycle arrest in G0/G1 phases, -promotion of apoptosis	synergistic	[126]
SAHA and clarithromycin (CAM) + bortezomib (BZ)	TNBC	MDA-MB-231	-enhancement of ER-stress-mediated cell death, -induction of apoptosis, -increase in *CHOP* and *GADD153* gene expression	synergistic	[127]
SAHA and tumor necrosis factor related apoptosis inducing ligand (TRAIL)	TNBC	BALB/c nude mice implanted with MDA-MB-468 TRAIL-resistant cells	-inhibition of tumor growth, metastasis and angiogenesis	synergistic	[128]
SAHA and soluble CD137 receptor	TNBC	MDA-MB-231	-increase in cytotoxicity	synergistic	[129]

**Table 4:** Vorinostat (SAHA) in clinical trials (N/A*—*not analyzed).

Clinical Trial	Clinical Trial Number	Drug and Therapy	Type of BC/Condition	Phase	Status	References
Olaparib in combination with vorinostat in patients with relapsed/refractory and/or metastatic breast cancer	NCT03742245	olaparib + vorinostat	breast cancer, metastatic breast cancer	I	recruiting	[155]
Carboplatin and nab-paclitaxel with or without vorinostat in treating women with newly diagnosed operable BC	NCT00616967	carboplatin + paclitaxel (albumin-stabilized nanoparticle formulation) + vorinostat or placebo	BC	II	active, not recruiting	[156]
Pembrolizumab and tamoxifen with or without vorinostat for the treatment of estrogen receptor positive breast cancer	NCT04190056	Pembrolizumab + tamoxifen + vorinostat	anatomic stage IV breast cancer AJCC v8, prognostic stage IV breast cancer AJCC v8	II	active, not recruiting	[157]
Trial for locally advanced breast cancer using vorinostat plus chemotherapy	NCT00574587	vorinostat + paclitaxel + trastuzumab + doxorubicin + cyclophosphamide and surgery	breast cancer	I, II	completed	[158]
HDAC inhibitor vorinostat (SAHA) with capecitabine (Xeloda) using a new weekly dose regimen for advanced breast cancer	NCT00719875	vorinostat	advanced breast cancer	I	completed	[159]
Ixabepilone and vorinostat in treating patients with metastatic breast cancer	NCT01084057	vorinostat + ixabepilone	male breast cancer, recurrent breast cancer, stage IV breast cancer	I	completed	[160]
Phase I–II study of vorinostat, paclitaxel, and bevacizumab in metastatic breast cancer	NCT00368875	vorinostat + paclitaxel + bevacizumab	male breast cancer, stage IIIB breast cancer, stage IIIC breast cancer, stage IV breast cancer	I, II	completed	[161]
Vorinostat in treating patients with stage IV breast cancer receiving hormone therapy	NCT01720602	vorinostat + anastrozole + letrozole + exemestane and radiation	male breast cancer, recurrent breast cancer, stage IV breast cancer	N/A	completed	[162]
Vorinostat in treating patients with stage IV breast cancer receiving aromatase inhibitor therapy	NCT01153672	vorinostat and radiation + anastrozole + letrozole + exemestane	male breast cancer, recurrent breast cancer, stage IV breast cancer	N/A	completed	[163]
Vorinostat in treating women who are undergoing surgery for newly diagnosed stage I–III breast cancer	NCT00262834	vorinostat and conventional surgery	breast cancer, stage I breast cancer, stage II breast cancer, stage III breast cancer	II	completed	[164]
Vorinostat and trastuzumab in treating patients with metastatic or locally recurrent breast cancer	NCT00258349	vorinostat + trastuzumab	breast cancer, male breast, cancer recurrent breast cancer, stage IIIB breast cancer, stage IIIC breast cancer, stage IV breast cancer	I, II	completed	[165]
Vorinostat in treating women with ductal carcinoma in situ of the breast	NCT00788112	vorinostat and neoadjuvant therapy and therapeutic conventional surgery	breast cancer	I	completed	[166]
Phase II trial of SAHA & tamoxifen for patients with breast cancer	NCT00365599	vorinostat + tamoxifen	breast cancer	II	completed	[167]
GCC 0845:vorinostat and lapatinib in advanced solid tumors and advanced breast cancer to evaluate response and biomarkers	NCT01118975	vorinostat + lapatinib	breast cancer, neoplasm, metastasis	I, II	terminated	[168]
A study of vorinostat and tamoxifen in newly diagnosed breast cancer	NCT01194427	vorinostat + tamoxifen	stage I breast cancer, stage II breast cancer, stage III breast cancer, invasive breast cancer	II	terminated	[169]
Suberoylanilide hydroxamic acid in treating patients with progressive stage IV breast cancer	NCT00132002	vorinostat	male breast cancer, recurrent breast cancer, stage IV breast cancer	II	terminated	[170]
Reversing therapy resistance with epigenetic-immune modification	NCT02395627	tamoxifen + vorinostat + pembrolizumab	breast neoplasms	II	terminated	[171]
A clinical trial of oral suberoylanilide hydroxamic acid (SAHA) in patients with relapsed or refractory breast, colorectal and non-small cell lung cancer (0683-011)	NCT00126451	MK0683 + vorinostat	breast cancer	II	terminated	[172]
Vorinostat before surgery in treating patients with triple-negative breast cancer	NCT01695057	vorinostat and therapeutic conventional surgery	stage II breast cancer, stage IIIA breast cancer, triple-negative breast cancer	N/A	withdrawn	[173]
Clinical trial of SAHA in patients with breast cancer	NCT00416130	vorinostat	breast cancer	I, II	unknown	[174]

## Data Availability

Not applicable.
